# Emergent Properties from Three-Dimensional Assemblies of (Nano)particles in Confined Spaces

**DOI:** 10.1021/acs.cgd.4c00260

**Published:** 2024-04-17

**Authors:** Emanuele Marino, R. Allen LaCour, Thomas E. Kodger

**Affiliations:** † Department of Physics and Chemistry, Università degli Studi di Palermo, Via Archirafi 36, Palermo 90123, Italy; ‡ Chemical Sciences Division, Lawrence Berkeley National Laboratory, Berkeley, California 94720, United States; ¶ Physical Chemistry and Soft Matter, Wageningen University and Research, Stippeneng 4, 6708WE Wageningen, The Netherlands

## Abstract

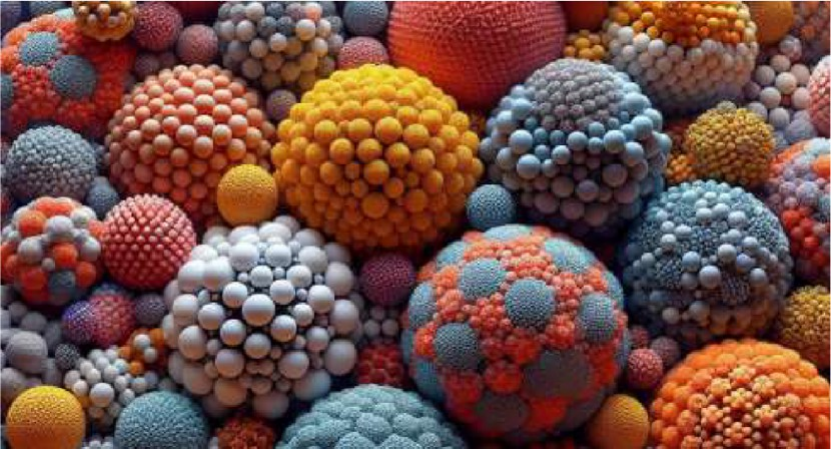

The assembly of (nano)particles into compact hierarchical structures yields emergent properties not found in the individual constituents. The formation of these structures relies on a profound knowledge of the nanoscale interactions between (nano)particles, which are often designed by researchers aided by computational studies. These interactions have an effect when the (nano)particles are brought into close proximity, yet relying only on diffusion to reach these closer distances may be inefficient. Recently, physical confinement has emerged as an efficient methodology to increase the volume fraction of (nano)particles, rapidly accelerating the time scale of assembly. Specifically, the high surface area of droplets of one immiscible fluid into another facilitates the controlled removal of the dispersed phase, resulting in spherical, often ordered, (nano)particle assemblies. In this review, we discuss the design strategies, computational approaches, and assembly methods for (nano)particles in confined spaces and the emergent properties therein, such as trigger-directed assembly, lasing behavior, and structural photonic color. Finally, we provide a brief outlook on the current challenges, both experimental and computational, and farther afield application possibilities.

## Abstract 2

Assembling superparticles: bringing together (nano)particles into higher order structures, new properties emerge not found in their building blocks.

## 1 Introduction

The assembly of simple building blocks into hierarchical structures represents a useful tool to enable the emergence of complex properties.^1−8^ These properties can be exploited to carry out otherwise difficult or impossible tasks. The vibrant colors of birds and beetles,^9,10^ algae photoprotection,^11^ or the impressive camouflaging abilities of chameleons^12^ and certain cephalopods^13^ highlight the ability of nature in exploiting assembly to improve the chances of survival for species that lack other defense mechanisms. In research, the assembly of simple building blocks into larger structures allows the construction of artificial materials with properties designed from the bottom-up that are more than the sum of the constituents. This approach can lead to materials that are superior in their structural strength and lightness, that show fade-free coloration by reflecting or absorbing specific colors of light and that can respond to specific external triggers by altering their properties in reversible and irreversible fashions.

The choice of the building block material is crucial in designing artificial materials from the bottom up. In its designs, nature uses building blocks that are readily available in the living organism, such as crystals of guanine in the case of chameleons. Artificial designs should include building blocks that are well-defined in their physical and chemical properties, and easy to synthesize and to assemble. Colloidal (nano)particles satisfy these requirements: (1) Their synthesis is well established, leading to well-defined sizes and shapes; (2) their composition can be tuned across the entire periodic table of elements, leading to a vast tunability in physiochemical properties; and (3) their surface chemistry allows for numerous options for functionalization, a versatile tool to design interactions with the surrounding medium and other particles. In particular, colloidal crystalline nanoparticles, or nanocrystals, are versatile building blocks for artificial materials. These nanocrystals combine a robust and well-defined inorganic structure of the core, responsible for the interaction with external electromagnetic stimuli, with a flexible organic structure of the ligand shell, responsible for the interaction with the surrounding medium. Other common building block options are inorganic (silica, titania) or polymeric colloidal particles.

**Figure 1:**
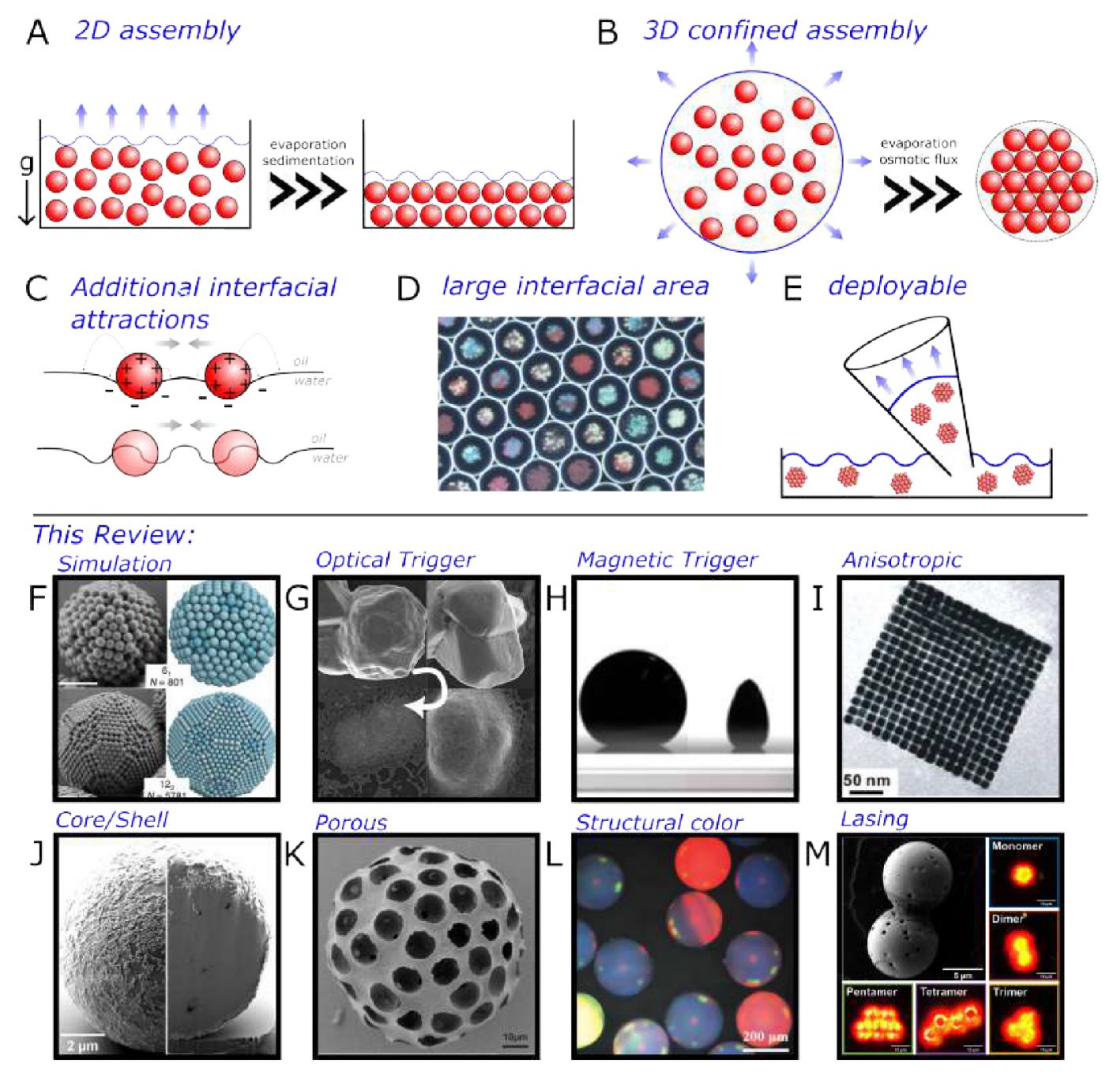
Assembly of (nano)particles. (A) Conventional 2D assembly through deposition and evaporation. (B) Confined 3D assembly discussed in this review. (C) Additional interparticle forces arising from the presence of an interface. (D) Confined assembly creates large interfacial areas.^31^ (E) Superparticles can be pipetted, transferred, and deployed. Topics discussed in this review: (F) Confined geometry simulation,^32^ (G) light triggered confined assembly,^33^ (H) magnetically triggered confined assembly,^34^ (I) anisotropic superparticles formed during confined assembly,^35^ (J) core/shell superparticles,^7^ (K) porous superparticles,^36^ (L) superparticles which exhibit structural coloration,^37^ and (M) superparticles with show lasing.^7^ Panel D is adapted with permission from ref (31). Copyright 2018 John Wiley and Sons. Panel F is adapted with permission from ref (32). Copyright 2018 Springer Nature. Panel G is adapted with permission from ref (33). Copyright 2023 The American Chemical Society. Panel H is adapted with permission from ref (34). Copyright 2019 The American Chemical Society. Panel I is adapted with permission from ref (35). Copyright 2012 The American Chemical Society. Panel J is adapted with permission from ref (7). Copyright 2022 The American Chemical Society. Panel K is adapted with permission from ref (36). Copyright 2018 The American Chemical Society. Panel L is adapted with permission from ref (37). Copyright 2019 John Wiley and Sons. Panel M is adapted with permission from ref (7). Copyright 2022 The American Chemical Society.

The interactions between colloidal particles govern their assembly into superstructures. For example, making the particles mutually attractive by functionalizing their surfaces with electric charges of opposite signs can induce assembly.^14,15^ However, irrespective of the interactions, increasing the density (volume fraction) of the colloidal dispersion through evaporation or sedimentation results in particle assembly; see Figure 1A. The fine details of the interparticle interactions, the rate of densification, and the topology of the system determine the final structure, which is generally a glass (disordered) or a crystal (ordered).

The dimensionality of this final structure also plays an important role in the emergent properties. Researchers first explored 2D interfacial assembly exploiting interfacial capillary forces and the experimental method, Langmuir–Blodgett, to controllably increase the density of particles.^16,17^ Few researchers have utilized 3D assembly in aerosols and glassware.^18,19^ 2D assemblies have the advantage of their ease of preparation and compatibility with existing “sandwich” device structures, such as those of photodetectors, solar cells, and light-emitting diodes; see Figure 1A. Nevertheless, 2D assemblies are limited in transferability, as they are typically confined to their substrate. Furthermore, the rate of production of 2D assemblies is usually low, as these must be prepared and optimized on a sample-by-sample basis by coating processes such as spin- or dip-coating. These techniques usually impose fast evaporation rates for the dispersing solvent, imposing kinetic constraints on the path toward equilibrium structures. Increasing the dimensionality of assemblies reverses the disadvantages of 2D assemblies while adding new functional features.

3D assemblies often result from the presence of a physical template imposing a specific topology during assembly. A simple template may be constituted by a well in which a particle dispersion is densifying as a result of solvent evaporation, resulting in multiple 2D assembled (nano)particle layers.^20,200^ Recently, emulsion droplets have been used as a 3D template for 3D assemblies.^3,7,8,21−26^ The controlled and complete removal of solvent from emulsion droplets filled with a dispersion of colloidal (nano)particles results in their controlled densification and assembly into spherical structures; see Figure 1B. These structures behave as larger colloidal particles, albeit they are constituted of smaller colloidal particles. In the literature, these structures are described by a compound noun including a preposition (“supra” or “super”) indicating the presence of a higher order within the structure and a simple noun (“particle” or “crystal”). The preposition “supra” directly hints at the term “supramolecular chemistry”,^27^ while “super” is consistent with the term “superlattice”,^28^ widely used in the nanocrystal community. The noun “particle” is used more generally in the literature, while “crystal” specifies the presence of an ordered internal structure.^29^

The formation of 3D assemblies is shaped by more features than 2D assemblies, contributing to a richer phase space for the resulting structures. The presence of a curved interface can influence the assembly process, as colloidal particles with a sufficiently large surface area may adsorb to the interface to decrease the energy of the system while introducing new interparticle attraction, such as electric field-dependent interfacial deformation and contact line undulations; see Figure 1C, top and bottom, respectively.^30^ This is the case for colloidal microparticles, resulting in particle-stabilized liquid droplets (Pickering emulsions) or (armored) gas bubbles. Furthermore, the presence of a large interfacial area increases the rate of mass transfer between droplets and their surrounding medium, allowing for efficient droplet densification and more controlled kinetics as compared to 2D assemblies, as displayed by the technique of droplet microfluidics; see Figure 1D.^31^ Another crucial advantage of 3D assemblies is the ease with which they can be transferred to different environments through simple techniques such as pipetting; see Figure 1E. For example, a superparticle capable of optically sensing and reporting on its local environment can be transported to a nearby target of interest to detect changes over time or in response to specific external triggers.

In this review, we illustrate recent advances in the emergent properties of 3D assemblies of (nano)particles in confined spaces. We begin by introducing some of the theoretical background and computer simulations behind particle assembly and the role of confinement on superparticle formation (Figure 1F). We proceed by illustrating different external triggers that can induce particle assembly, such as light and static magnetic fields (Figure 1G–H). The interplay between anisotropic building block shapes and interparticle interactions can yield anisotropic superparticle shapes (Figure 1I). Furthermore, the interplay between incompatible building block shapes and sizes can lead to phase separation, resulting in core/shell and porous structures (Figure 1J–K). Finally, we review the relationships between the morphological and structural characteristics of superparticles and their optical response, leading to structural color and lasing (Figure 1L–M).

## 2 Results and Discussion

### 2.1 Insights from Theory and Simulation

The properties of the (nano)particle building blocks and their environment largely determine the final structure that results from assembly. As the microscopic sizes of these systems make it challenging to directly observe the assembly process, computer simulations have played an important role in elucidating how the final structure forms. In particular, molecular dynamics simulations reproduce the self-assembly process for specified interparticle and particle-interface interactions and have thus proven a rich source of insight. In this section, we summarize many of these efforts along with the experimental systems they are meant to correspond to.

Perhaps the simplest example of assembly in confinement is that of (nano)particles or nanocrystals under the spherical confinement of liquid droplets. Due to its simplicity, this is also the most investigated system in simulations. Both experiments and simulations reveal a size dependence of the confining droplets, which causes deviations from the tendency of spherical particles to form a face-centered-cubic (FCC) crystal structure.^38^ For droplets containing under ≈10^5^ particles, de Nijs et al. found that the particles can form arrangements with icosahedral symmetry.^22^ While icosahedral symmetry is incompatible with long-range order, in finite systems this structure can have a lower free energy than the competing FCC phase.^32,39^ A similar behavior was observed in binary nanocrystal systems, in which the bulk MgZn_2_ phase is distorted into an icosahedral cluster,^26^ indicating that the influence of confinement may be quite general. Furthermore, Wang et al. found that particle organization may be influenced by how close the number of confined particles is to a “magic number”, for which the free energy of the confined particles becomes particularly low, and that being an off- or on-magic number can play a major role in determining which defects are present.^32,39,40^ Mbah et al. later found that entropy-stabilized dodecahedral structures could also form and obeyed a different set of magic numbers from the icosahedral structures.^41^ We note that this preference for icosahedral and dodecahedral arrangements was found in the experimental assembly of nanocrystals, but simulations were able to reproduce this behavior using only hard-sphere-like particles confined by a hard interface, and thus entropy appears to be the driving force for these structures.

Recent simulations have retained the spherical confinement but moved away from spherical building blocks. In 2016, Teich et al. examined the packing of small numbers of polyhedra (<60) in spherical confinement, finding that many polyhedra pack into layers of optimal spherical codes.^42^ Particularly dense packing arrangements were also found to occur at magic numbers.^42^ In 2018, Wang et al. demonstrated that rounded cubes inside spherical confinement display, as a function of degree of rounding, a transition from an FCC-like phase to a simple cubic structure with aligned orientations, in agreement with experiment.^43^ In 2022, Skye et al. investigated the self-assembly of confined tetrahedral particles, finding that the curvature of the droplet promoted the formation of specific structural motifs that could propagate further into the droplet.^44^ As the size of the droplet dictates the curvature, changing the size of the droplet tuned the structure. Also in 2022, Wang et al. found that the shape parameters of nanoplatelets in confinement influence their behavior, leading to a variety of phases like discotic or liquid crystal phases.^40^ In all these examples, entropy was the driving force for self-assembly.

The cases described, where entropy is primary driving force, apply primarily to particles with weak interparticle attractive forces. However, frequently interactions between nanocrystals may be quite attractive.^45,46^ In this case, the presence of emulsion droplets appears to exert a smaller influence on the resulting bulk structure. Nonetheless, the emulsion droplet system provides a convenient platform for 3D self-assembly and *in situ* X-ray scattering, facilitating easier comparisons between simulation and experiment. Both Montanarella et al. and Marino et al. found that their nanocrystals crystallized at low densities, which they could only reproduce in simulations by adding interparticle attraction.^24,25^ Later, Marino, Lacour, et al. found that the short-range attractive forces can dramatically accelerate the self-assembly of certain binary nanocrystal superlattices.^8^ In both cases, the confining droplet appeared to have limited influence on the superlattice structure.

Lastly we note that, while molecular dynamics simulations have revealed a wealth of information about structural formation, further progress is still needed to elucidate often complex experimental results, as discussed in the following sections. Most of the examples described above focus on simpler systems where the interparticle interactions are well-known. A key challenge to overcome is to increase our understanding of interactions in more complex systems, which require significant and continued joint efforts of experiment and simulation.

### 2.2 Externally Triggered Self-Assembly

#### 2.2.1 Light-Activated Self-Assembly

The specific response of nanocrystals to external stimuli, such as electromagnetic fields, can lead to triggerable colloidal interactions. These interactions can induce nanocrystal assembly into complex superstructures that reflect the characteristics of the external trigger. In this section, we discuss the growth of complex superstructures with a morphology that derives directly from the details of the interaction between nanomaterials and light.^47−50^

**Figure 2:**
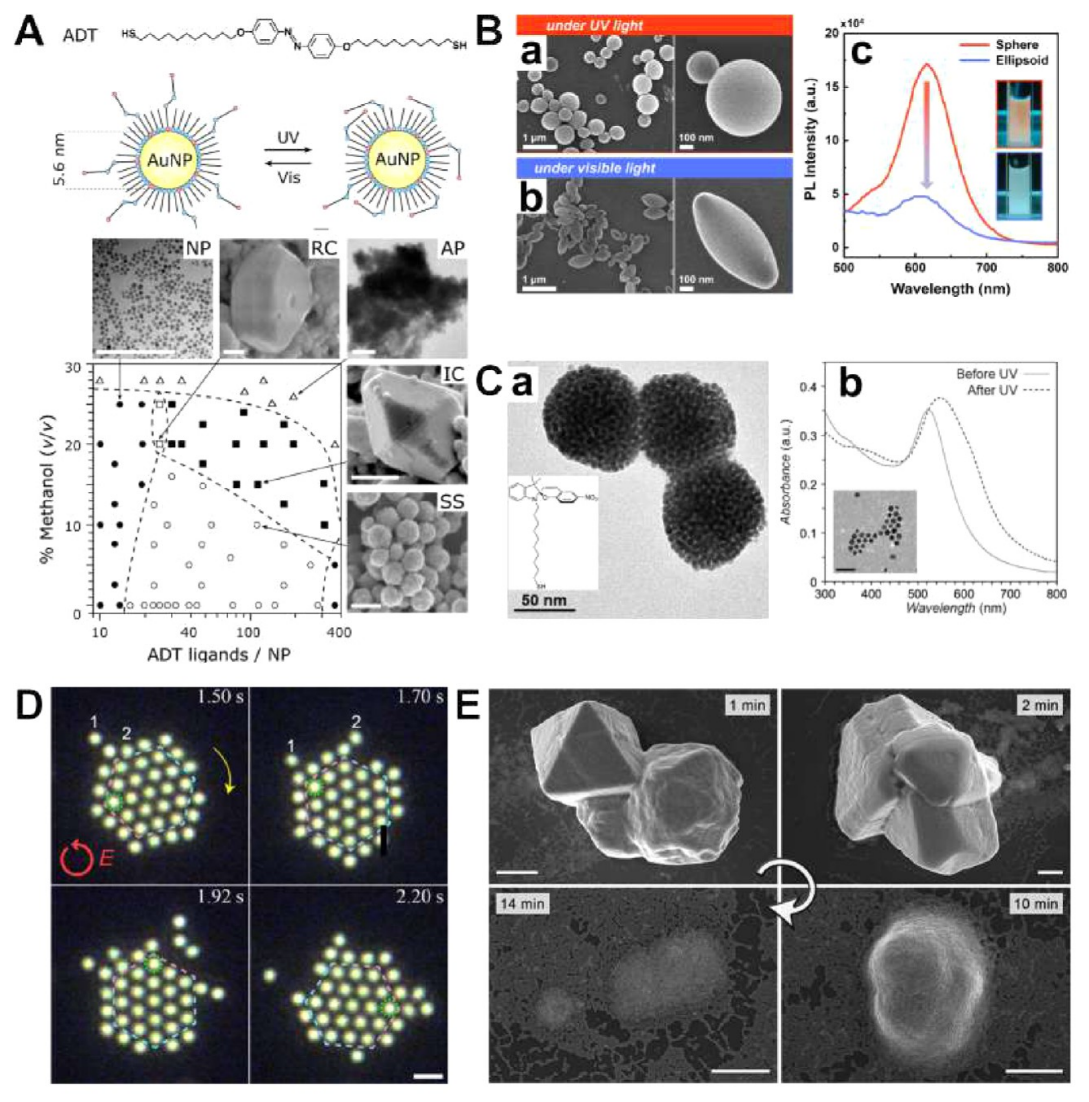
Light-activated self-assembly. (A) (Top) Ligand used for light-activated self-assembly and structural changes in the nanoparticle (NP) ligand corona upon photoexcitation with ultraviolet and visible light. (Bottom) Phase diagram and SEM images of suprastructures obtained by light-activated self-assembly for photoswitchable ligands density and composition of the dispersing solvent. Legend: NP, dispersed NPs; RC, light-reversible 3D superlattices; AP, amorphous precipitate; IC, irreversible 3D superlattices; SS, superparticles. (Scale bars are 100 nm.)^1^ (B) SEMs (low and high magnifications) of PS10k-*b*-P4VP10k superparticles prepared with (a) ring-opened form of the surfactant and (b) ring-closed form of the surfactant. (c) Photoluminescence (PL) spectra of spherical (orange) and ellipsoidal (blue) PS10k-*b*-P4VP10k superparticles. Insets: fluorescence optical images of the superparticle suspension during assembly.^51^ (C) (a) TEM image of three superparticles of 5.5 nm Au NPs functionalized with the thiolated spiropyran, as shown as inset. (b) Extinction spectra of 5.5 nm Au NPs in toluene before and after photoexcitation with ultraviolet light. Inset shows the TEM of the NPs, scale bar indicates 20 nm.^52^ (D) Optical micrographs of optically tweezed Ag nanocrystal superlattices (laser intensity = 3 × 10^9^ W*/*m^2^). The dashed hexagon represents the boundary between the central area and the edge of the superlattice, while the color of the hexagon and the dashed circle indicate lattice orientation. Scale bar indicates 1 μm.^53^ (E) Representative SEMs of the nanocrystal superlattices of Au-TMA/NBTS after ultraviolet exposure for the indicated times (scale bars indicate 0.5 μm).^33^ Panel A is adapted with permission from ref (1). Copyright 2007 The American Association for the Advancement of Science. Panel B is adapted with permission from ref (51). Copyright 2021 The American Chemical Society. Panel C is adapted with permission from ref (52). Copyright 2016 The Royal Society of Chemistry. Panel D is adapted with permission from ref (53). Copyright 2020 The American Chemical Society. Panel E is adapted with permission from ref (33). Copyright 2023 The American Chemical Society.

In an important contribution, Klajn et al. examined the role of ligand photoisomerization on the formation of superstructures from Au nanocrystals; see Figure 2A.^1^ Upon photoexcitation with ultraviolet light, the isomerization of the azobenzene-based ligands induces the formation of electric dipoles on the nanocrystal surface.^54^ These dipoles are responsible for nanocrystal assembly through dipole–dipole interactions. The superstructures assemble readily, creating a confined environment that can trap molecules within, beating diffusion.^55^ The composition of the ligand corona is crucial in determining assembly kinetics. Very low and very high surface coverage by the azobenzene-based ligand do not result in assembly, since in the former case not enough dipoles are excited, while in the latter case steric hindrance hinders isomerization (NP, closed circles in Figure 2A). Instead, at intermediate surface coverages the photoinduced dipole–dipole interactions results in the formation of nanocrystal superparticles (SS, open circles in Figure 2A). The authors leveraged solvophobic interactions by varying the composition of the solvent from pure toluene to a toluene/methanol mixture. This increases the magnitude of attractive interactions between nanocrystals, resulting in the formation of reversible 3D nanocrystal superlattices (RC, open squares in Figure 2A). The magnitude of the interactions was further increased by increasing the coverage of photoresponsive ligands on the nanocrystal surface, resulting in the formation of irreversible 3D nanocrystal superlattices (IC, closed squares in Figure 2A). Increasing the magnitude of the interparticle interactions further resulted in the formation of amorphous aggregates, as nanocrystals become kinetically trapped before reaching their minimum-energy configurations (AP, open triangles in Figure 2A). Importantly, since these changes are related to the ligand choice, they should be generalizable to different nanocrystal core compositions.

Photoactivated phase change can also be driven in block copolymer superparticles; see Figure 2B. Kim et al. were able to form block copolymer superparticles by dissolving block copolymer (PS-bP4VP) in chloroform and preparing a chloroform-in-water emulsion, subsequently allowing the chloroform to evaporate.^51^ The authors stabilized the droplets by using a photoresponsive version of the popular surfactant DTAB based on spiropyran. The spiropyran group isomerizes between its less hydrophilic (ring-closed) form under visible light and the more hydrophilic (ring-opened) form under ultraviolet light. Therefore, performing the assembly under visible or ultraviolet light affects droplet stabilization and self-assembly, as the block copolymer exposes the block favored by the surfactant toward the interface. When performing the assembly under visible light, the ring-opened form of DTAB would favor interaction with P4VP, leading to the formation of onion-like spherical superparticles characterized by an outer layer of P4VP; see Figure 2Ba. Instead, when performing the assembly under ultraviolet light, the ring-closed form of DTAB shows no preference between both PS and P4VP blocks, leading to ellipsoidal superparticles; see Figure 2Bb. These structural and morphological changes also bring optical changes. Indeed, the surfactant bound to the superparticles can either be emissive or not, depending on morphology as the ring-open configuration is conjugated, while the ring-closed configuration is not; see Figure 2Bc.

When used as ligands, the photoactivated isomerization of spiropyran can also affect the assembly of nanocrystals. Kundu et al. studied this effect by functionalizing Au nanocrystals with thiolated spiropyran ligands, see structure in the inset of Figure 2Ca.^52^ The photoexcitation of the spiropyran with 365 nm light results in the opening of the ring, leading to the more hydrophilic ring-opened form resulting in the prompt aggregation of Au nanocrystals into spherical superparticles, Figure 2C, left. The authors were able to follow the assembly and redispersion kinetics in detail by studying the effects of mesostructure formation on the extinction spectra of Au nanocrystals, Figure 2Cb. Before photoexcitation, the extinction spectrum for 5.5 nm Au nanocrystals shows a sharp peak around 530 nm that is typical for these systems. After 40 s of exposure to ultraviolet excitation, the extinction spectrum shows a broadening and a red-shift of the plasmonic resonance to 560 nm, possibly accompanied by the development of an additional band further in the red at 640 nm: As Au nanocrystals come closer in space, their plasmonic resonances begin to interact to emulate the effect of larger nanocrystals.

The role of light on the assembly of nanocrystals can vary, as shown by the interesting contribution of Han & Yan.^53^ The authors were able to trap large (up to 101 nanocrystals) arrays of large (diameter = 150 nm) Ag and Au nanocrystals by an optical trap generated by circularly polarized light while observing the process through dark-field microscopy; see Figure 2D. When the laser intensity reaches 2.2 × 10^9^ W*/*m^2^, the nanocrystals assemble to form 2D hexagonal superlattices. The stability of the nanocrystals within the superlattices is controlled by the laser intensity and the proximity of the nanocrystals to the edge of the superlattice, where thermal fluctuations have a larger influence. Interestingly, when increasing the laser intensity further, the nanocrystal superlattice begins to rotate around its center of mass in a direction opposite to the electric field, resulting in a negative optical torque. The authors attribute the presence of a negative optical torque to the discrete rotational symmetry of the system; that is, each nanocrystal rotates independently thanks to the transfer of angular momentum from the circularly polarized beam to the nanocrystal, while the superlattice rotates as a whole.

So far, we have spoken of systems where light plays an active role in inducing assembly. Recently, Wang et al. designed a system where the opposite is true: nanocrystal superlattices irreversibly assembled through molecular “glues” can be redispersed by photocleavage.^33^ Au nanocrystals passivated by positively charge trimethylammonium (TMA) groups are colloidally stable in water thanks to their electrostatic interactions. However, the addition of a molecule endowed with three or more negative charges, such as nitrobenzyltrisuccinate (NBTS), leads to the colloidal destabilization of the nanocrystals, resulting in their assembly; see Figure 2E. However, the exposure of NBTS to ultraviolet excitation (365 nm) leads to its transformation into nitrosobenzyldisuccinate (NBDS) and succinate dianions. The two negative charges of NBDS are unable to compensate for the positive charges of TMS, resulting in the gradual redispersion of Au nanocrystals; see Figure 2E.

#### 2.2.2 Magnetic-Field-Activated Self-Assembly

Nanocrystals responsive to magnetic fields open up multiple assembly configurations and superstructures,^56,57^ resulting in a modulation of the magnetic response.^58^ This is especially true when additional elements are introduced to the assembly playground, such as the geometric confinement imposed by a droplet,^59^ or additional interactions with interfaces,^2^ other nanocrystals,^60^ or ligands.^61^ In this section, we highlight some of the works that exploited magnetic interactions to induce nanocrystal assembly.

**Figure 3:**
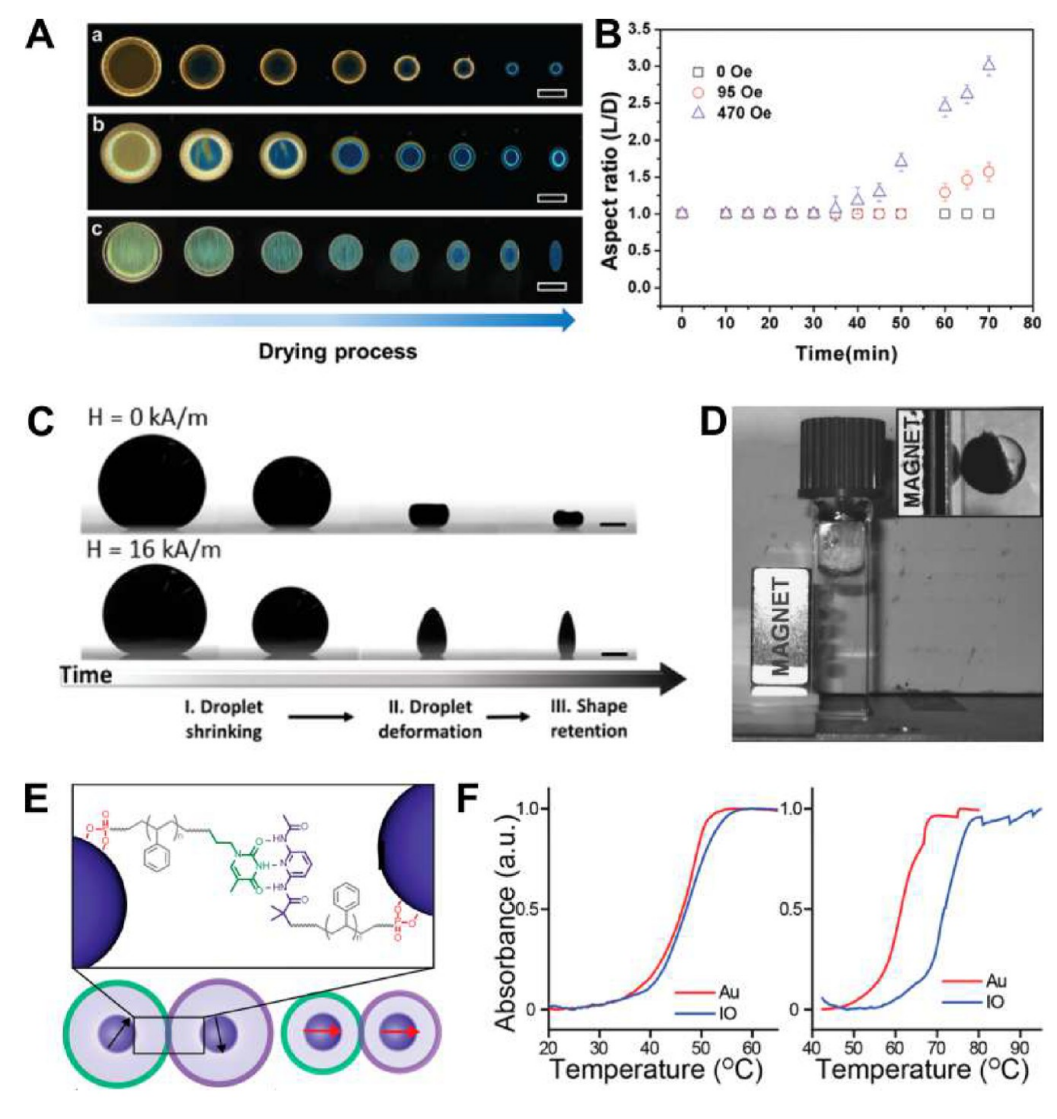
Magnetic-field-activated self-assembly. (A) Dark-field optical micrographs showing the evolutions of droplets at different drying stages: (a) absence of an external magnetic field, (b) under a horizontal magnetic field of 95 Oe, and (c) of 470 Oe. Scale bars indicate 100 mm. (B) Evolution of droplet aspect ratios with time corresponding to A.^62^ (C) Evolution of a droplet loaded with 3 wt % of Fe_3_O_4_ nanocrystals embedded in polystyrene during drying in the absence (top) and the presence (bottom) of an external magnetic field. Scale bars indicate 0.5 mm.^34^ (D) Magnetically anisotropic Janus superparticles; inset: magnified image of a single superparticle with the PNIPAm side attracted to a magnet.^63^ (E) Schematic of the experimental system composed of nanocrystal cores, polymer brush, and supramolecular binding groups that drive assembly. Assembly is directed via complementary hydrogen bonding moieties, strengthened via magnetic dipole coupling between aligned spins at short interparticle distances. (F) (Left) Melting profiles of 16 nm iron oxide nanocrystals functionalized with 13 kDa polymers and 15 nm Au nanocrystals functionalized with 14 kDa polymers. (Right) Melting profile of 16 nm iron oxide coated with 8 kDa polymers is shifted by 10 °C with respect to 15 nm Au nanocrystals coated with 9 kDa polymers.^64^ Panels A and B are adapted with permission from ref (62). Copyright 2019 The Royal Society of Chemistry. Panel C is adapted with permission from ref (34). Copyright 2019 The American Chemical Society. Panel D is adapted with permission from ref (63). Copyright 2009 John Wiley and Sons. Panels E and F are adapted with permission from ref (64). Copyright 2020 The American Chemical Society.

In an interesting demonstration, Liu et al. showed that magnetic fields can be used to direct the self-assembly of anisotropic magnetic nanocrystals into anisotropic superparticles.^62^ The authors use microfluidics to generate water-in-hexadecane droplets containing a dispersion of Fe_3_O_4_/SiO_2_ ellipsoidal nanocrystals (250 by 150 nm), as seen from the dark-field optical micrographs in Figure 3A. As water is removed from the droplet through evaporation, a solid superparticle is formed. Surface tension effects dominate over the anisotropic shape of the nanocrystal, leading to spherical superparticles with an aspect ratio of 1.0; see Figure 3A,a,b. This is no longer true when a magnetic field is applied to the droplet during evaporation: The droplet preserves the spherical shape during the first 30 min of evaporation but then starts to deviate leading to the formation of anisotropic superparticles with an aspect ratio of 1.6; see Figure 3Ab and B. The aspect ratio can be increased to 3.0 by increasing the magnitude of the magnetic field from 95 Oe to 470 Oe; see Figure 3Ac and B. The tendency of the magnetic nanocrystals to align their easy axis of magnetization with the field is contrasted by surface tension trying to minimize the surface per unit volume of the droplet.

However, this experiment is not scale-invariant. A similar experiment carried out at a 10-fold larger scale on a superamphiphobic substrate investigated the competition between magnetic effects and gravity. To do so, Hu et al. cast 5 μL water droplets loaded with Fe_3_O_4_ nanocrystals embedded in polystyrene particles and studied the evolution of droplet morphology during evaporation; see Figure 3C.^34^ In the absence of a magnetic field, the droplet displays an initial contact angle with the substrate of 161°, which gradually decreases to 150° as evaporation takes place. As the density increases, the droplet shows a clear tendency to collapse under its own weight, leading to anisotropic droplets with a pancake shape. The presence of a magnetic field parallel to the gravitational field leads to significant changes in the assembly. Initially, the droplet behaves similarly to the case of no magnetic field; however, as evaporation occurs, the droplet shows a stark change in shape from spherical to ellipsoidal, with the long axis of the ellipsoid parallel to the magnetic field. The authors attributed this sudden deformation to the formation of a solid shell in the droplet during assembly. Evaporation causes the particle accumulation to the shell, inducing stresses that can be promptly released by a buckling mechanism such as the one observed. The beautiful interplay of surface tension, gravitational, and magnetic forces leads to strongly anisotropic superparticles shapes.

One of the advantages of magnetic nanocrystals is the possibility of directing the retrieval of superstructures by using magnetic fields. Indeed, in the case of nonmagnetic nanocrystals, separation and purification routes usually rely on solvophobic interactions, solvent removal, or sedimentation. Instead, in the case of magnetic nanocrystals, the application of a magnetic field is sufficient to separate the magnetically responsive components. Shah et al. drove the formation of Janus superparticles by generating water-in-oil emulsion droplets containing a solution of thermoresponsive pNIPAm microgels, acrylamide monomer, cross-linker, and photoinitiator; see Figure 3D.^63^ Upon heating, the microgels shrink and aggregate to one side of the droplet. Upon photoexcitation with ultraviolet light, the monomer polymerizes, generating solid Janus superparticles. When added, magnetic nanocrystals become jammed between the pNIPAm microgels, implementing an orthogonal functionality to the superparticles without disrupting their formation.

Advanced synthetic ligand design can mediate, reinforce, or disrupt magnetic interactions. In particular, Santos & Macfarlane have shown that the hydrogen bonds present between ligand terminations can increase the magnitude of the attractive interactions between neighboring nanocrystals.^64^ The authors designed a ligand consisting of a polymer brush terminated on one end by a phosphonate group bound to the nanocrystal surface and on the other end by hydrogen-bonding moieties; see Figure 3E. Since the hydrogen-bonding interactions are sensitive to temperature, nanocrystals terminated with such a ligand tend to assemble at room temperature but redisperse when increasing the temperature, as shown by the melting profile in Figure 3F. The assembly behavior is insensitive to the choice of nanocrystal core, since similarly sized Au and iron oxide cores show similar values of melting point (46–47 *°*C); see Figure 3F, left. However, the interactions between nanocrystal cores start playing a significant role when the size of the polymer is decreased from 13 to 14 kDa to 8–9 kDa, requiring higher temperatures to redisperse with melting points of 72 and 61 °C for iron oxide and Au respectively; see Figure 3F, right. This interesting observation highlights the role of magnetic interactions on the assembly: When the nanocrystals are sufficiently close, the spins of neighboring magnetic nanocrystals are able to couple, leading to an increase in the magnitude of the attractive interactions with respect to magnetically nonresponsive counterparts.

### 2.3 Superparticle Morphologies

#### 2.3.1 Anisotropic Superparticles

**Figure 4:**
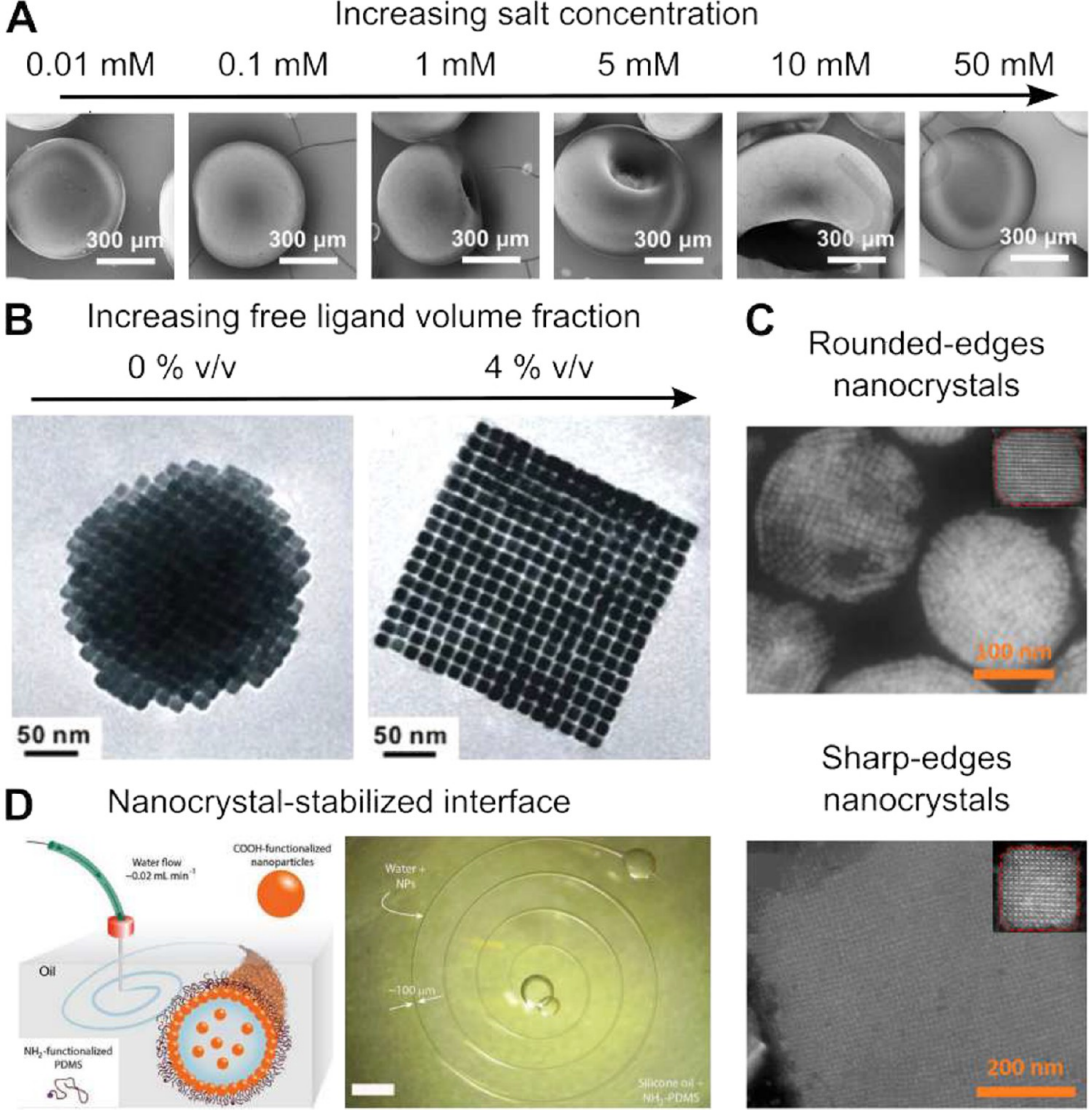
Anisotropic superparticles. (A) Scanning electron micrographs of superparticles obtained by the evaporation of aqueous droplets with different NaCl concentrations containing a dispersion of polystyrene spheres 440 nm in diameter at a volume fraction of 8%.^65^ (B) Transmission electron micrographs of superparticles consisting of 11 nm nanocubes of Fe_3_O_4_ assembled in the presence excess oleate ligands.^35^ (C) Scanning transmission electron micrographs of superparticles consisting of CsPbBr_3_ nanocrystals characterized by rounded (top, 14 nm diameter) and sharp (bottom, 10 nm diameter) edges.^66^ (D) (Left) Schematic of the stabilization of aqueous wires in a silicone oil by hydrophilic 20 nm SiO_2_ nanocrystals. (Right) Optical micrograph of a nanocrystal-stabilized aqueous spiral in silicone oil.^67^ Panel A is adapted with permission from ref (65). Copyright 2022 Elsevier B.V. Panel B is adapted with permission from ref (68). Copyright 2012 The American Chemical Society. Panel C is adapted with permission from ref (66). Copyright 2020 The American Chemical Society. Panel D is adapted with permission from ref (67). Copyright 2018 John Wiley and Sons.

Obtaining anisotropically shaped superparticles is not straightforward as surface tension imposes a spherical shape to maximize the volume-to-surface ratio of droplets. However, by clever design of the experimental system, it is possible to circumvent this limitation. In this section, we summarize recent results to synthesize spatially anisotropic superparticles. Controlling the interactions between colloidal particles can be effective in modifying the final shape of superparticle.^65^ Interparticle interactions such as electrostatic, (ligand) depletion, geometric free volume due to particle shape, and interfacial adsorption can induce anisotropic superparticles, while these types of interaction have no inherent anisotropy, as shown in Figure 4.

##### 2.3.1.1 Mechanical Stability of the Air/Liquid Interface

When using charge-stabilized polystyrene colloids, Liu et al. observed that increasing the concentration of electrolyte within the droplet resulted in flattening and buckling of the resulting superparticles; see Figure 4A. Interestingly, at low (<1 mM) and high (>10 mM) salt concentrations, the superparticles appear spherical, while at intermediate (1–10 mM) salt concentration the superparticles appear anisotropic, featuring one prominent dimple at 1 mM salt concentration that develops in a donut-like shape at 10 mM. The authors justified these results by the decrease in Debye screening length due to the increase in salt concentration, resulting in the irreversible aggregation of colloids during droplet evaporation. At low salt concentrations the colloidal behavior is that of repulsive particles slowly densifying by evaporation, while at high salt concentration the particles immediately aggregate in the bulk of the solution; neither of these regimes affects the mechanical stability of the air/liquid interface. However, the partial screening of surface charges that occurs at intermediate salt concentrations results in the self-assembly of nanoparticles directly at the air/liquid interface. Further densification of the droplet causes the mechanically strengthened interface to deform, resulting in anisotropic superparticle shapes. Similar results were observed by Velev et al. when decreasing the volume fraction of the particle within water-in-fluorinated-oil droplets, highlighting the development of dimples at an initial volume fraction of 10%, and the transition to donut-like shapes below 4%.^3^

##### 2.3.1.2 Ligand-Based Interactions

In the case of lipophilic colloidal particles, it is possible to obtain anisotropic shapes of superparticles by exploiting the interplay between interparticle interactions and surface tension. Wang et al. observed that the densification of chloroform-in-water droplets consisting of a dispersion of oleate-stabilized iron oxide nanocubes gave rise to spherical superparticles, shown in Figure 4B.^35^ However, the addition of 4% v/v oleic acid to the oil phase of the emulsion resulted in the cubic shape of the resulting superparticles. The authors justified these observations by the increased magnitude of the attractive interparticle interactions resulting from the interdigitation of oleate chains, although depletion interactions between nanocrystals are also likely to play a role.^69,70^ Furthermore, the authors argued that oleic acid likely results in a decrease in surface tension of the liquid/liquid interface, accommodating more easily the anisotropic shapes of the growing crystallites during densification. Interestingly, the crystalline structure adopted by the nanocrystals within the superparticles appeared insensitive to the concentration of free oleic acid, suggesting that the shape of the nanocrystal and their magnetic interactions represented the main driving forces behind the choice of the crystalline lattice.

##### 2.3.1.3 Shape-Based Interactions

The shape of the colloidal (nano)particles acting as building blocks for assembly plays an important role in determining the ultimate shape of the superparticles. Subtle differences in nanocrystal shapes can result in major changes at the end of the assembly process. Recently, Tang et al. observed that preparing superparticles from aged CsPbBr_3_ perovskite nanocubes resulted in spherical superparticles, while using freshly synthesized samples led to cubic assemblies, shown Figure 4C.^66^ The authors observed that aging the nanocubes under ambient conditions resulted in the smoothing of their initially sharp corners, explaining their results. Wang et al. studied the interplay between spherical confinement and particle shape on the self-assembly of rounded cubes in a dedicated study.^71^ The authors found that sharp cubes strongly align to form simple-cubic superstructures. Instead, rounded cubes assemble into icosahedral clusters with strong local orientational correlations driven by their nonisotropic shape. More recently, combining perovskite nanocrystals with sharp corners with other nanocrystal shapes has emerged as a succesful strategy to nucleate novel superlattice types.^201,202,203^

Assembling nanocrystal building blocks with larger anisotropic ratio leads further exacerbates the interplay between nanocrystal shape and spherical confinement. van der Hoeven et al. assembled porous-silica-coated Au nanorods by using a water-in-oil emulsion to yield porous superparticles.^72^ The use of a silica shell with a thickness comparable to the nanorod diameter tended to conceal the dipolar shape of the nanorods, resulting in little orientational correlation between adjacent nanorods and noncrystalline assembled structures. However, using a thin silica shell resulted in higher orientational correlation between adjacent nanorods and smectic-like assembly. When optimized, the anisotropic shape of nanorods can influence the final shape of the superparticles. As Wang et al. showed, assembling CdSe/CdS nanorods using an oil-in-water emulsion results in the formation of barrel-shaped superparticles with morphologies dependent on the nanorod aspect ratio and the nanorod-to-superparticle size ratio.^68^ This behavior is similar to that of nanoplatelets, with the difference that in this case the spherical shape of the superparticle is barely affected due to the tendency of the nanoplatelets to stack parallel to the interface during densification to fill the remaining volume.^73^

##### 2.3.1.4 Particle-Interface Interactions

When the liquid/liquid interface is stabilized by surfactants, nanocrystals assemble under the constraint of the receding interface but are unaffected by the energetics of the interface itself. This changes dramatically when the nanocrystals act as interface-stabilizers. In the absence of other surfactants, nanocrystals adsorb to the interface to minimize the free energy of the system, also known as Pickering emulsions. This behavior introduces additional mechanical properties for the interface, resulting in the buckling behavior discussed earlier. Cui et al. showed that applying an electric-field to Pickering emulsions stabilized by charged particles resulted in anisotropic droplets.^74^ Interestingly, after removal of the dispersed phase of the emulsion, the shape was preserved, resulting in anisotropic superparticles. Pickering emulsions can bring other interesting morphological effects. Extruding a nanocrystal dispersion in water into highly viscous silicone oil results in the stabilization of the water/oil interface by the nanoparticles, allowing printing of highly anisotropic shapes, such as spirals (Figure 4D) and letters.^67,75^

#### 2.3.2 Core/Shell Superparticles

The internal architecture of superparticle can be modified in several ways to achieve a morphology characterized by distinct compositions close to and away from the surface. In this section, we discuss core/shell superparticles, how they can be obtained through different strategies, and why they are interesting for a number of applications from drug delivery to lasing.^76,77^

##### 2.3.2.1 Double Emulsions

**Figure 5:**
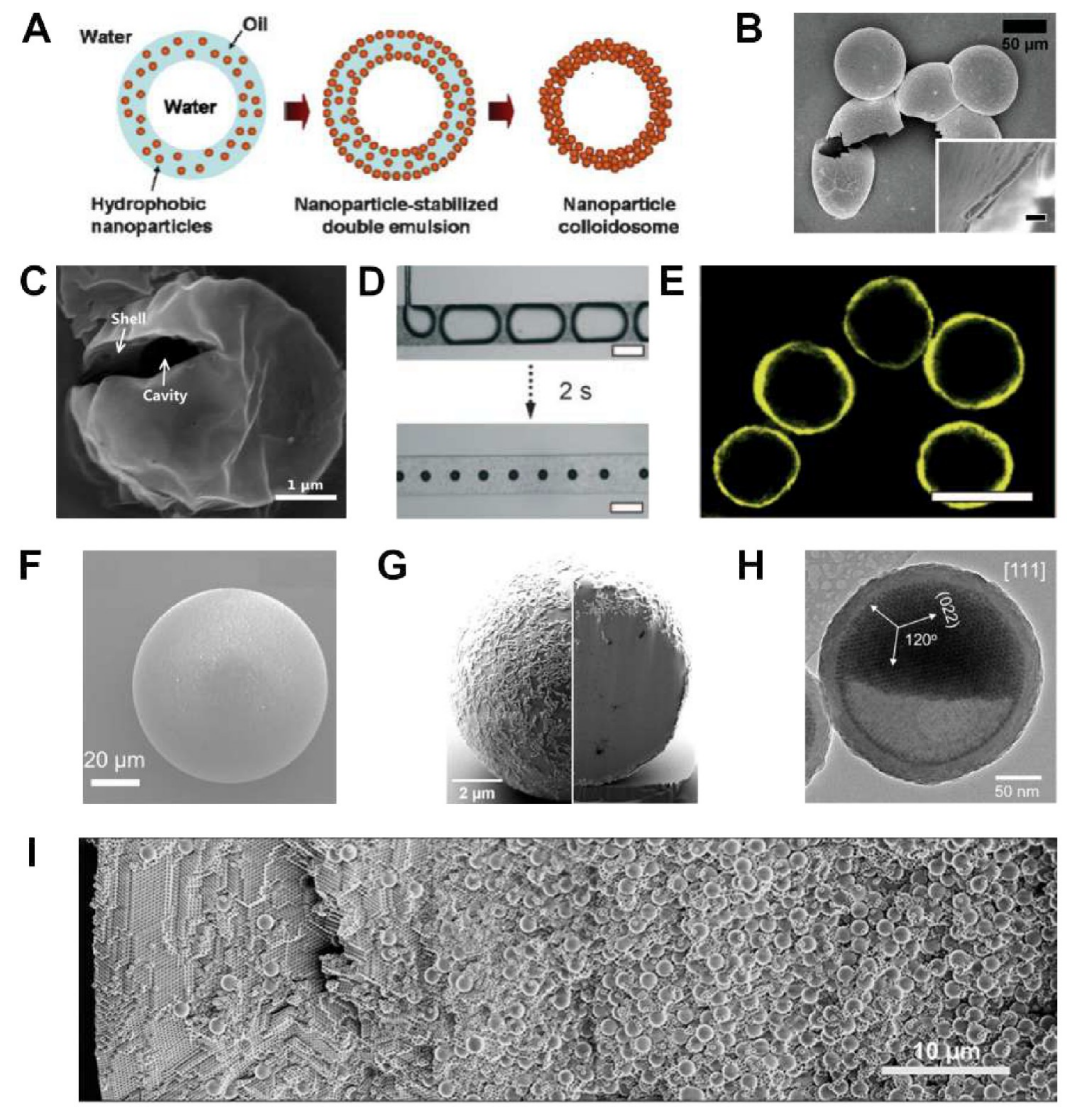
Core/shell superparticles. (A) Schematic for the formation of colloidosomes from nanocrystal-stabilized water-in-oil-in-water double emulsions. (B) SEM image of poly(d,l-lactic acid)/SiO_2_ dried colloidosomes. Inset shows the cross-section of the broken shell (scale bar indicates 500 nm).^78^ (C) SEM image of acetylated dextran colloidosomes. The broken colloidosome reveals the cavity and shell thickness.^79^ (D) (Top) Optical micrographs of CO_2_ bubbles generated in an aqueous dispersion of 3.5 μm polystyrene microparticles at a volume fraction of 1.5% w/w. (Bottom) The bubbles quickly decrease in size to lead to the formation of spherical colloidosomes. Scale bars indicate 200 μm. (E) The colloidosomes become fluorescent when using polystyrene particles loaded with CdSe/ZnS nanocrystals. Scale bar indicates 100 μm.^80^ (F) SEM image of a SiO_2_ microsphere uniformly coated with CdSe/CdS@Cd_1–*x*_Zn_*x*_S core/shell nanoplatelets.^81^ (G) SEM image of a core/shell nanocrystal superparticle consisting of PbS/CdS spheres and NaGdF_4_ disks before (left) and after (right) focused-ion beam milling, revealing the phase separation of nanocrystals.^7^ (H) TEM image of a half-full colloidosome viewed along the [111] zone axis of the Fe_3_O_4_ nanocrystal superlattice.^82^ (I) Overlay of SEM image of the cross section of a superparticles consisting of 338 nm and 1430 nm polystyrene colloidal particles at initial volume fractions of 2.5% and 5.5%, respectively.^83^ Panels A and B are adapted with permission from ref (78). Copyright 2008 John Wiley and Sons. Panel C is adapted with permission from ref (79). Copyright 2015 The American Chemical Society. Panels D and E are adapted with permission from ref (80). Copyright 2009 John Wiley and Sons. Panel F is adapted with permission from ref (81). Copyright 2022 The American Chemical Society. Panel G is adapted with permission from ref (7). Copyright 2022 The American Chemical Society. Panel H is adapted with permission from ref (82). Copyright 2016 The American Chemical Society. Panel I is adapted with permission from ref (83). Copyright 2019 The American Chemical Society.

For a specific application, it may be desirable to confine nanocrystals to a specific region of the superparticle, such as in proximity of the surface, while leaving the inner volume empty to be used to load other components. This can be done by preparing double emulsion droplets consisting of two interfaces.^84^ In a important contribution, Lee & Weitz showed that, when confining silica nanoparticles to the intermediate phase of water-in-oil-in-water emulsions, the evaporation of the oil phase leads to the formation of a solid silica shell (Figure 5A,B).^78^ This type of superparticle with a capsule-like structure takes the name of *colloidosome*.^4^

##### 2.3.2.2 Particle-Interface Interactions

Colloidosomes can also be produced using less advanced droplet architectures. Vasiliauskas et al. used oil-in-water emulsions to generate colloidosomes with well-defined polymer-based shells (Figure 5C).^79^ The authors chose an oil with an intermediate solubility in water such as dimethyl carbonate (up to 13.9%, v/v) to achieve superparticle formation by the diffusion of oil in water without affecting droplet formation. Upon droplet densification, the polymer dissolved in the oil phase showed an affinity to the interface, resulting in the formation of hollow superparticles. The choice of a polymer that degrades under low-pH conditions, such as acetylated dextran, allowed the authors to show that colloidosomes can be effective at releasing encapsulated drugs on demand.

Rather than exploiting the affinity for the interface of a species dispersed or dissolved in the dispersed phase of the emulsion, Park et al. demonstrated the fabrication of colloidosomes by exploiting the affinity for the interface of particles dispersed in the continuous phase.^80^ After generating CO_2_ bubbles in an aqueous dispersion of 3.5 μm polystyrene particles, the authors observed that the size of the bubbles quickly decreased over time as a result of the diffusion of CO_2_ in water; see Figure 5D. However, the shrinking of bubbles was not indefinite and resulted in the formation of colloidosomes as the result of the adsorption of polystyrene particles at the H_2_O/CO_2_ interface. The solid shell can be endowed with fluorescent capabilities by functionalizing the polystyrene particles with emissive CdSe/ZnS nanocrystals (Figure 5E).

##### 2.3.2.3 Particle–Particle Interactions

The use of particles with different sizes can be effective in relegating specific physical functions to certain regions of a superparticle. A prototypical example can be the coating of a single silicon dioxide microsphere with colloidal CdSe/CdS/Cd_1–*x*_Zn_*x*_S nanoplatelets, as shown in Figure 5F. Such superparticle design allows the photoluminescence emission by nanoplatelets to couple with the whispering-gallery modes of the microsphere, effectively resulting in a microresonator and leading to lasing.^81^ A similar strategy can be implemented at commensurate scales of nanocrystals by exploiting differences in sizes and shape by mixing ≈4 nm PbS/CdS spheres and ≈60 nm NaGdF_4_ disks, resulting in the separation of the nanocrystal components within a superparticle.^7^ Imaging the superparticle before and after milling by focused ion beam demonstrates the separation of disks to the proximity of the superparticle surface, as seen in Figure 5G. While the interactions between different nanocrystal shapes can promote phase separation, exotic results can be obtained also by using a single nanocrystal shape. Yang et al. achieved interesting superparticles morphologies by exploiting the interactions between self-assembled and dispersed Fe_3_O_4_ nanocrystals, see Figure 5H.^82^ The authors first generated colloidosomes stabilized by the self-assembly of nanocrystals at the oil/water interface, followed by further structural stabilization through the growth of a silica shell. Upon the gradual removal of the oil from the colloidosomes, the nanocrystals still dispersed inside nucleated heterogeneously at the interface. As a result, the final superparticle features the external structure of a colloidosome with an internal volume partially filled by a crystalline superstructure. The interactions between nanocrystals with the same shape but different in size can also drive phase separation. Liu et al. showed that the smaller polystyrene colloids tend to occupy the portion of the superparticle closer to the interface, while the larger colloids occupy the central volume of the superparticle; see Figure 5I.^83^

#### 2.3.3 Porous Superparticles

The assembly of nanocrystals under conditions of increasing confinement often leads to dense and isotropic superstructures that minimize the surface per unit volume.^8^ However, the generation of high surface area superstructures, such as porous superparticles, enables important advantages such as sequestration, storage, and directed release of cargo, as well as increasing the reactivity of the superparticle for chemical reactions taking place on its surface.^85^ Over the past few years, a number of strategies have emerged to generate porous, high-surface area superparticles.^86−90^

##### 2.3.3.1 Porous Building Blocks

**Figure 6:**
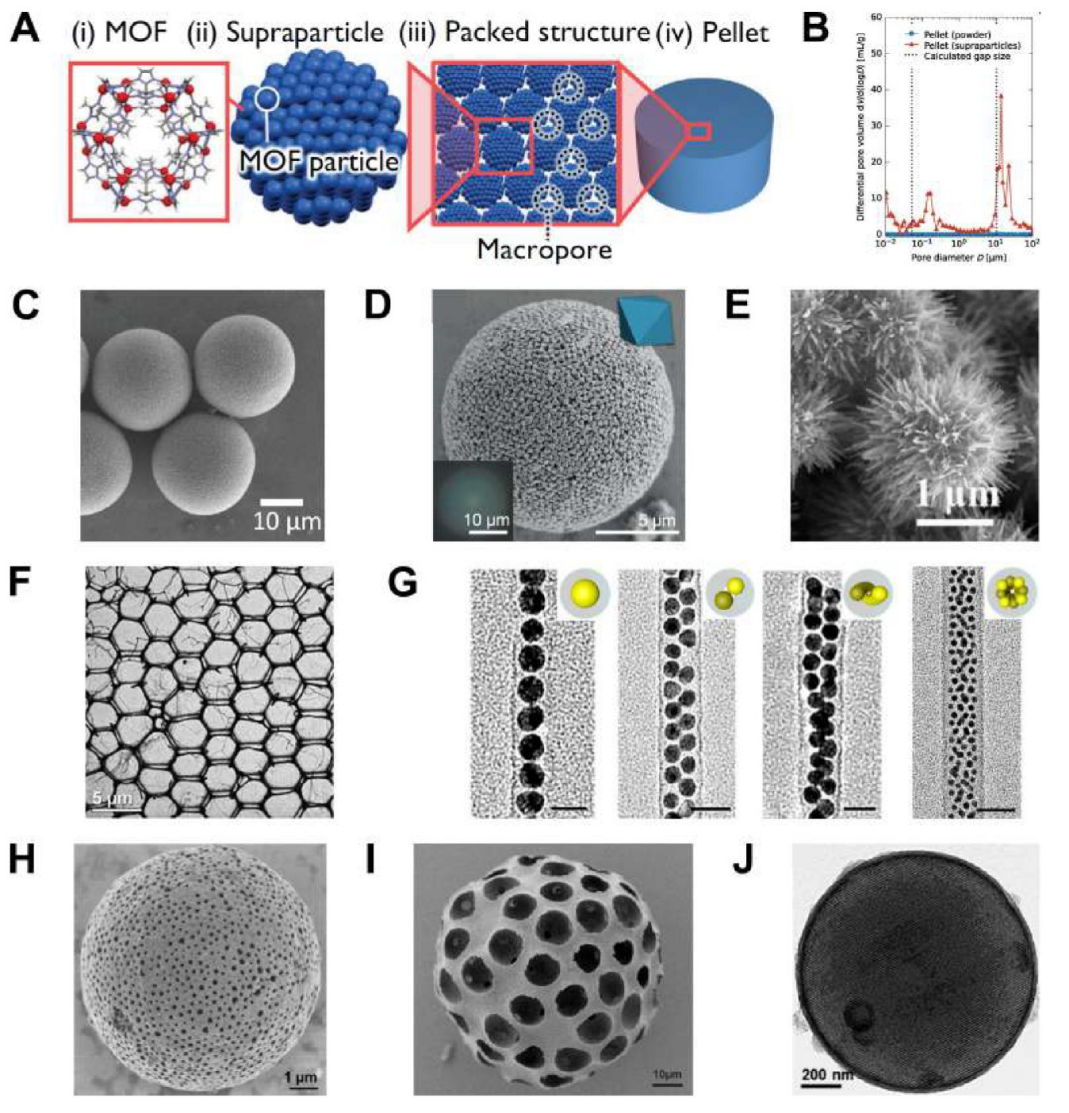
Porous superparticles. (A) Fabrication of hierarchically structured materials based on superparticles. ZIF-8 metal–organic framework (MOFs) particles form superparticles, which are further assembled into macroscopic pellets with structural hierarchy of pores.^91^ (B) Pore size distribution of pellets of ZIF-8 particles and superparticles.^91^ (C) SEM image of ZIF-8 superparticles.^91^ (D) SEM image of (UiO-66) MOFs superparticles which exhibit structural coloration (optical microscopy images in inset).^92^ (E) SEM image of complex nanostructured hedgehog superparticles from CdS assembled at unity water-to-ethylenediamine volume ratio and 160 °C for 20 h.^93^ (F) Network structure formed by a 1:1 mixture of Au and CdSe/CdS/ZnS nanocrystals passivated with a dendritic ligand drop-cast from chloroform at 15 mg/mL.^94^ (G) TEM image of Au nanocrystal assemblies with diverse structures at different diameter ratio (γ) between carbon nanotube hosts and Au nanocrystal guests: γ = 1.1, 1.5, 1.8, 2.3. Scale bars indicate 20 nm.^95^ (H) SEM image of porous CoFe_2_O_4_ superparticles.^5^ (I) SEM image of a porous silica SP after removal of the polystyrene nanoparticles by calcination.^36^ (J) TEM image of hybrid micromesoporous graphitic carbon superparticles.^96^ Panels A, B, and C are adapted with permission from ref (91). Copyright 2023 John Wiley and Sons. Panel D is adapted with permission from ref (73). Copyright 2022 John Wiley and Sons. Panel E is adapted with permission from ref (93). Copyright 2021 The American Chemistry Society. Panel F is adapted with permission from ref (94). Copyright 2022 The American Chemistry Society. Panel G is adapted with permission from ref (95). Copyright 2021 The American Chemistry Society. Panel H is adapted with permission from ref (5). Copyright 2022 The American Chemistry Society. Panel I is adapted with permission from ref (36). Copyright 2018 The American Chemistry Society. Panel J is adapted with permission from ref (96). Copyright 2017 The American Chemistry Society.

A straightforward strategy to develop porosity in superstructures consists of choosing building blocks that are already porous. As porous crystalline materials varying in size from the nano- to the microscale, metal–organic frameworks (MOFs) represent an ideal choice. Fujiwara et al. executed on this plan by assembling 190 nm zeolitic imidazolate framework-8 (ZIF-8) into 20–80 μm spherical superparticles by using microfluidic droplets.^91^ After removal of the dispersed phase, the superparticles were assembled into higher order macroscopic pellets, see Figure 6A. Interestingly, the pellets feature two sets of pore sizes, 2 orders of magnitude apart, highlighting the pores present between MOF particles (150 nm micropores) as well as those between superparticles (14 μm macropores); see Figure 6B,C. The micropores also lead to a spatial modulation of the refractive index of the material, resulting in an enhanced interaction with light through diffraction. Specific wavelengths are reflected by the superparticle, leading to structural color, a property resulting directly from the controlled porosity of the superstructures; see Figure 6D.^92^ In the future, building blocks may benefit from combining the porosity of MOFs with the response versatility of nanocrystals. Dedicated synthetic designs have already shown the possibility of embedding nanocrystals within the structure of MOFs.^97^

##### 2.3.3.2 Particle–Particle Interactions

When starting with nonporous building blocks, exploiting colloidal interactions can be key to produce high-surface area superstructures. Tang et al. showed that polydisperse CdS nanocrystals can assemble to form corrugated particles visually resembling a hedgehog; see Figure 6E.^93^ The high-surface area of the hedgehog particles results in interesting properties, such as their omnidispersibility^98,99^ and their peculiar interaction with light.^100,101^ By combining experiments and simulations, the authors depict a complex assembly pathway controlled by the energetic balance between van der Waals attraction and electrostatic repulsion. Within the polydisperse ensemble of Cd*S* nanocrystals, the largest nanocrystals assemble first as a consequence of their stronger van der Waals attraction forces, forming a superparticle core. Consequently, nanocrystals of smaller and smaller sizes undergo oriented attachment to the core, leading to the formation of the high-surface area spikes.

Following similar principles, two-dimensional porous structures can also be synthesized; see Figure 6F. Elbert, Vo, et al. show that the use of ligands that can cross-link nanocrystals can be crucial.^94^ When a dispersion of nanocrystal dries, the density of nanocrystals tends to increase in the proximity of the evaporation front, ultimately resulting in their assembly. The process of internanocrystal cross-linking reduces nanocrystal mobility, ultimately resisting displacement. The interplay between cross-linking and evaporation can lead to the formation of nanocrystal networks. More recently, this concept was extended to 3D by using droplets microfluidics.^205^ The authors combined two nanocrystal types with a strong tendency to phase-separate within rapidly densifying emulsion droplets, resulting in highly porous superparticles.

##### 2.3.3.3 Porous Host Design

The generation of high-surface area superstructures can result from the deliberate choice of a porous host structure. Zhang et al. followed this strategy by coassembling inorganic nanocrystals within carbon nanotubes.^95^ The authors found that when the nanocrystals have an effective diameter smaller than the internal diameter of the nanotube, the nanocrystals have a tendency to fill the nanotube. The interplay between 1D confinement and the size ratio between nanocrystals and tube internal diameter results in several possible structures of different symmetries, as shown in Figure 6G. Instead, blocking the entrance to the nanotube cavity molecular species results in the assembly of nanocrystals to form a layer on the outer surface of the nanotube.

A porous host structure can also be generated during nanocrystal assembly. Yang et al. have shown the combination of large water/oil ratios and low shear rates can lead to the formation of complex double emulsions of water-in-oil-in-water where a large droplet of oil-in-water contains many smaller water droplets.^5^ The removal of oil leads to nanocrystal self-assembly into superparticles, while the removal of water leads to the formation of pores within the superparticle; see Figure 6H. The possibility of tuning the porosity of nanocrystal superparticles, increasing the amount of surface area available, opens up new avenues to their use as anode materials for batteries.^5,102^

Increasing the porosity of a nonporous superparticle is possible by the selective removal of a fraction of the assembled nanocrystals. This idea was initially introduced for 2D crystalline assemblies of nanocrystals of two different sizes, commonly referred to as binary nanocrystal superlattices.^103,104^ When the composition of nanocrystals is chosen such that chemical conditions that completely dissolve one population of nanocrystals leave the other population untouched, nanoporous structures are generated.^105^ Moving from 2D to 3D requires sufficient molecular transport of the etchant and the etched species; a few successful examples have already emerged. Egly et al. coassembled 300 nm silica and 10 μm polystyrene particles in a water-in-oil emulsion template.^36^ After assembly, the authors proceeded with calcination to remove the polystyrene spheres, leaving the silica superparticles microporous; see Figure 6I.

The generation of fully organic porous superparticles can also be conjectured by the selective removal of inorganic nanocrystal components through chemical treatments. Zheng et al. achieved this goal by first assembling oleate-stabilized iron oxide nanocrystals using an oil-in-water emulsion template.^96^ After assembly, hydrogen chloride is used as an etchant to fully dissolve the inorganic core of the nanocrystal, leaving the structure fully organic and nanoporous; see Figure 6J. This porous structure is then able to act as host for lithium–sulfur^96^ and sodium-ion^106^ batteries, as well as electrocatalyst for oxygen reduction reaction.^107^

### 2.4 Structure–Property Relationships

#### 2.4.1 Structural Color

The assembly of nanocrystals under confined conditions has opened up a wide range of opportunities to develop new structure-properties relations. The assembly of larger, colloidal nanoparticles, with radii on the order of the wavelength of visible light (150–500 nm), has also been explored toward a specific structure property relation: structural (physical) color. For millennia, nature has been utilizing structural color to produce vibrant, fade-free, iridescent colors across multiple *classes* of animals, namely, *insecta*, in beetles (e.g., *Chrysochroa fulminans*) and butterflies (e.g., *Morpho didius*), algae (*Chondrus crispus*),^11^ and *aves*, birds (e.g., *Ara ararauna*). Often, natural structural colors comprise an inverse opal structure which has been for nearly two decades the most common structure for researchers.^108^ In this section, we discuss recent progress in the use of droplets as a confinement strategy to develop new structural color superparticles while also permitting their scaled production.

Through the removal of the dispersed phase, as shown in Figure 1B, the particle volume fraction can increase while developing crystalline symmetries displaying macroscopic structure color. Additionally, if this volume fraction increases rapidly compared to the diffusion of the particle, disordered structural color may also form.^109^ Such structural color pigments have only recently been dispersed into other formulations, e.g. coating, to create dispersible structural color paint.^110,111^

**Figure 7:**
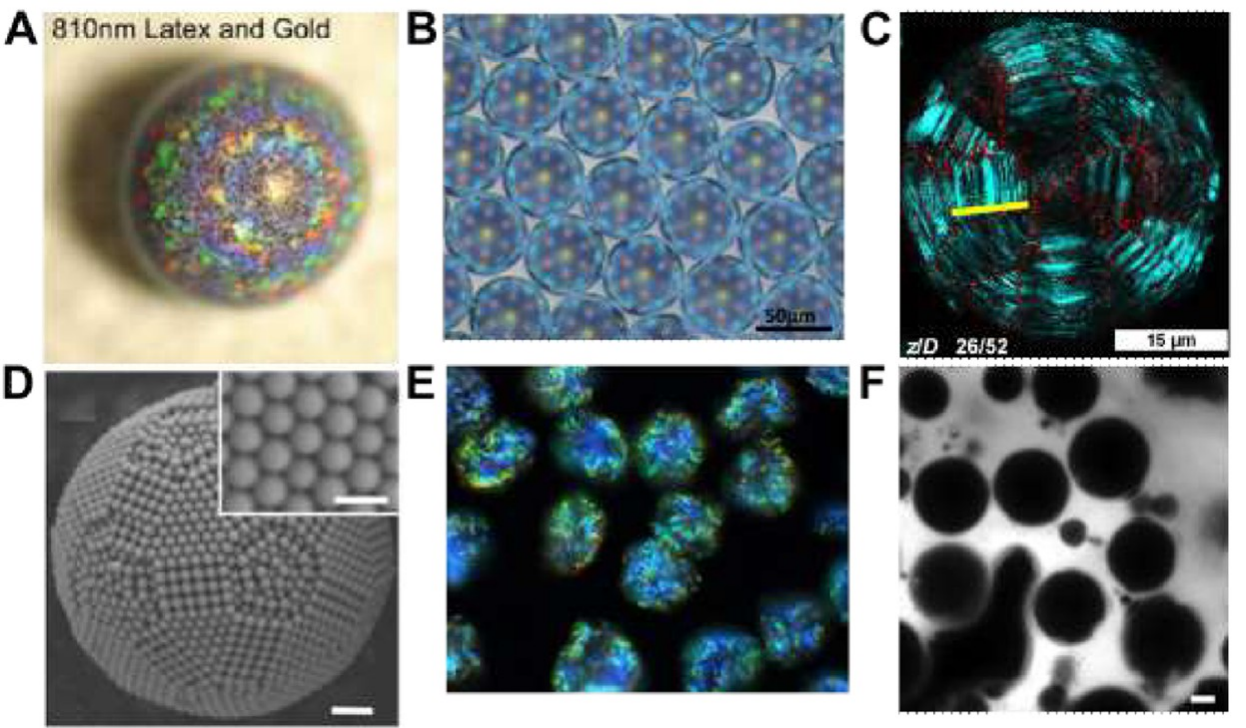
Structural color pigments. (A) 810 nm latex particles dried containing 0.21 wt % of 22 nm diameter Au nanocrystals (particle diameter is 50 μm).^112^ (B) A monolayer of microfluidically produced structural color spheres composed of 610 nm diameter latex particles.^6^ (C) Reflectance confocal microscopy (RCM) image of a structural color pigment at approximately the midplane of the particle, note the appearance of multiple crystalline domains.^115^ (D) SEM image of a structural pigment created with icosahedral cluster composed of 275 nm diameter particles (scale bar indicates 1 μm). Inset: A magnified SEM image of one of the hexagonal faces of the cluster. Scale bar indicates 200 nm.^117^ (E) Dark-field microscopy image of cellulose nanocrystal pigments dispersed in oil (refractive index 1.55).^111^ (F) Fluorescence microscopy image of silica-based inverse-opal structural pigments in a dried film of latex paint. Note: the fluorescence intensity is excluded from the round structural color particle; (scale bar indicates 10 μm).^110^ Panel A is adapted with permission from ref (112). Copyright 2008 John Wiley and Sons. Panel B is adapted with permission from ref (6). Copyright 2008 The American Association for the Advancement of Science. Panel C is adapted with permission from ref (115). Copyright 2021 The American Chemical Society. Panel D is adapted with permission from ref (117). Copyright 2020 The American Chemical Society. Panel E is adapted with permission from ref (111). Copyright 2022 Springer Nature Publishing Group. Panel F is adapted with permission from ref (110). Copyright 2019 John Wiley and Sons.

Before the use of a liquid–liquid confinement method, Velev et al. utilized superhydrophobic surfaces of Low Density PolyEthylene (LDPE) to spatially isolate a colloidal dispersion of polystyrene particles of diameters ranging from 320–1000 nm.^112^ Interestingly, they found that the incorporation of a low concentration (∼1 wt %) of 20–22 nm Au nanocrystals increased the observed reflectance and suppressed backscattering of “white” colors, shown in Figure 7A. Crucially, the superhydrophobic substrate allowed simple transport and deployment of these structurally colored superparticles. Similarly, structural color pigments were made by Kim et al. using the Leidenfrost effect for spatial confinement.^113^ Interestingly, these authors also incorporated carbon black nanoparticles to act as broad-band absorbers of incoherent multiply scattered light, enhancing the magnitude of the reflected color.

Droplet microfluidics offers a direct route for monodisperse droplet confinement creating monodisperse structural color pigments. Vogel et al. used microfluidics and dispersions of latex particles to create monodisperse “photonic balls”^6^ with the similar approach of adding Au nanocrystals,^112^ seen in Figure 7B. Kim et al. also used microfluidics to create monodisperse structural color double emulsion spheres which by weakening the interparticle repulsion created multiple crystal packings with colors spanning the visible spectrum.^37^ Crucial to achieving this color-spanning photonic structure is control over their crystallinity; structural color has also been used as a tool to characterize 3D structural information and heterogeneity.^114^ Other experimental approaches such as reflectance confocal microscopy^115^ as shown in Figure 7C, and focused-ion beam scanning electron microscopy (FIB-SEM)^6^ have been used to study the interior 3D structure of photonic pigments which has opened up new avenues of characterization and elucidated new understanding of structure–property relations in structural coloration. Additionally, other technologies than droplet microfluidics, such as inkjet printing, have been used to create structural color under confinement with low magnitude of angularly dependent reflection.^116^

Colloidal particle symmetry plays an important role in the magnitude of saturation of structural color. Moon et al. employed icosahedral symmetry of colloidal clusters to enhance color and reduce the radial, onion-like, ordering typically found in confined droplet geometries Figure 7D.^117^ Iridescence is also often found in natural structural color; spherical confinement has been recently explored by Yoshioka et al., who found that Bragg diffractions from different crystal planes played an essential role.^109^ Disordered colloidal glasses, with no long-range order, have been richly investigated for their noniridescent structural color with multiple experimental examples, while disordered structural color in the red remains challenging as was explored by Manoharan et al.^118^ In addition to spherical colloidal particles in confined geometries, composite and nonspherical particles have also been explored as structural color building blocks which may perturb the intrinsic crystallinity.^119^ Interestingly, Kim et al. found that using an absorbative nanoparticle of eumelanin can absorb the incoherent scattered light, thus enhancing the structural color saturation without affecting the underlying crystallinity.^120^

Recently, the use of structural color pigments created in confined geometries has greatly expanded toward real-world application concomitant with the development of improved color saturation and spectral specificity of individual pigment particles. The use of nonspherical, cellulose-based particles by Vignolini et al. has recently offered a commercially viable approach to structural color pigment based on biobased polymers, shown in Figure 7E.^111^ The textile industry also employs large amounts of pigments often suffering from fade; Shao et al. used inkjet printing to directly create structural color droplets on individual fibers.^121^ Larger scale production of structural color pigments have also been developed for paint films and coatings using a polydisperse emulsion and a sol–gel process.^110^ The resulting inverse-opal structure, when dispersed in a latex dispersion, retained refractive index contrast due to the expulsion of the film-forming latex particles by the pores of the structural pigment dimension, as seen in Figure 7F. In addition to films, coatings, and textile, structural color has also been explored as a counterfeiting technique, as the precise reflectance spectrum of the pigment is challenging if not impossible to mimic.^122^ Structural coloration through the addition of pigments created in confined geometries is still an emergent field with researchers pursuing multiple challenges. These include creating highly saturated colors, colors spanning the entire visible spectrum, composite colors (see Figure 6D), through mixing of multiple pigments, suppressing iridescence, and creating scalable processes based on renewable resources as the application possibilities are broad and require larger scale production beyond individual droplets-based approaches.

#### 2.4.2 Lasing

Recently, the interaction between nanocrystal superparticles and light has been subject of intense study. Classical electromagnetism (Mie theory) predicts the formation of hot spots of the electric field within dielectric objects commensurate with the wavelength. These resonances are known as *Mie resonances* and result in increased absorption and scattering cross sections at the resonant wavelengths. The spectral position of these resonances depends on particle size, shape, and refractive index contrast, leading to a morphology- and composition- dependent optical response that can be exploited for many applications. For instance, the light scattered by the particles may be used to increase the absorption efficiency of solar cells by inducing the trapping of light within the active medium, or may lead to pigment-free coloration (structural color). When the particles are larger than the wavelength of light and show rotational symmetry, the interaction between light and matter favors a different coupling mechanism. A symmetrical object with a diameter larger than the wavelength of light supports symmetrical optical modes in proximity of the object’s surface, known as *whispering-gallery modes*. Through these modes, light can become trapped near the surface of the particles. When the particle consists of a gain medium, the increased intensity of the electric field near the particle’s surface may induce population inversion and lasing even under conditions of low excitation fluence. The tunability in optical response of dielectric, semiconductor, and metallic nanocrystals, combined with the morphological tunability of superparticles, leads to a fascinating scientific playground to build assembled optical resonators operating in any range of frequencies. In this section, we discuss some advances in this direction.

**Figure 8:**
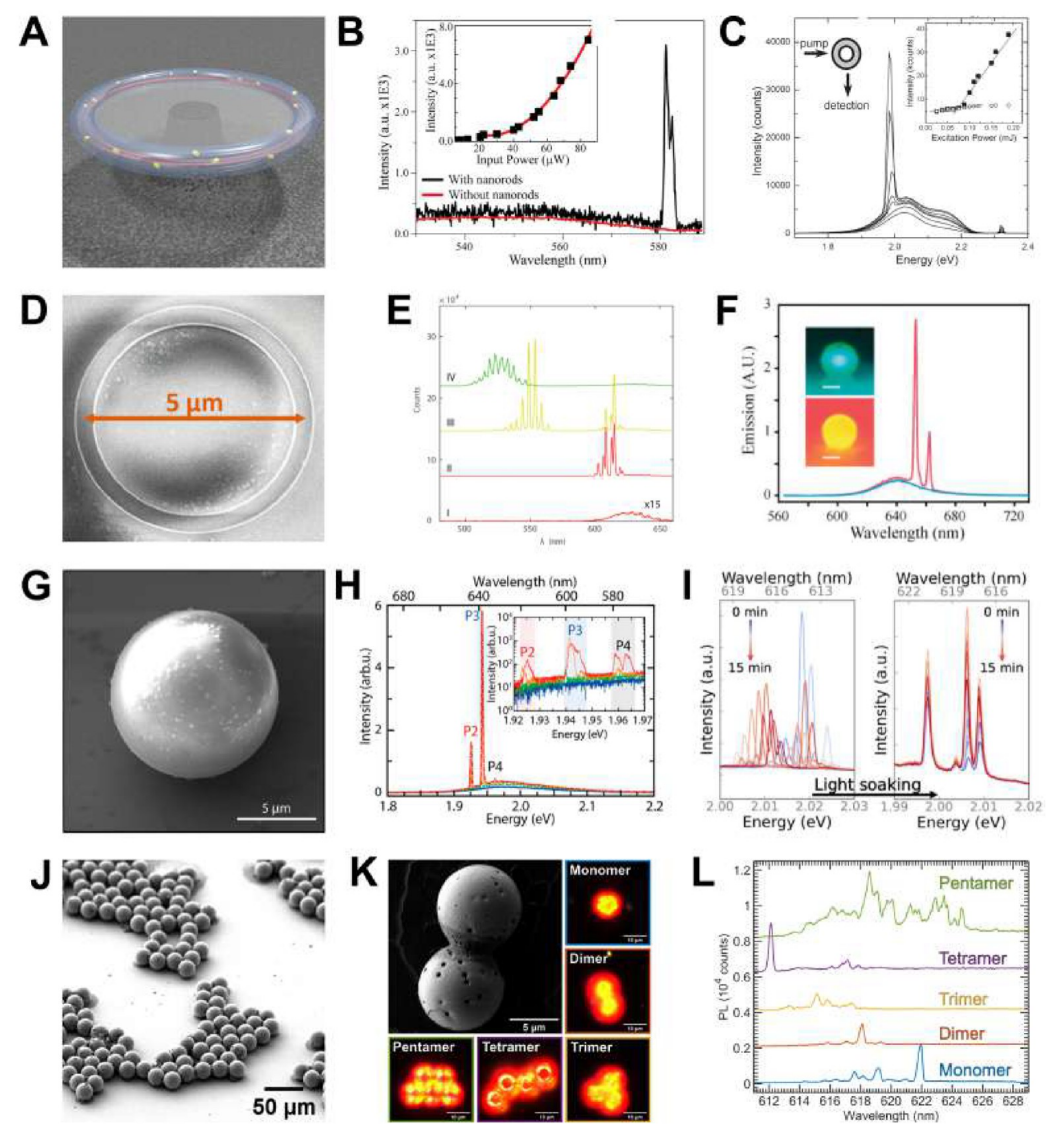
Lasing superparticles. (A) Rendering of a toroidal optical resonant cavity coated with Au nanorods.^123^ (B) Lasing spectra and threshold data (inset) of Au nanorod-coated microtoroid cavity. The quadratic relationship between the lasing intensity and the input power is due to the two-photon lasing mechanism.^123^ (C) Emission spectra for CdSe/ZnS nanorods loaded in the microcavity at different pump powers. (Inset) intensity of the lasing peak (filled squares) and the fluorescence peak (empty circles) versus the pump power.^124^ (D) SEM image of a CdSe/CdS/ZnS nanocrystal ring resonator with diameter and width of 5 μm and 500 nm, respectively.^125^ (E) Representative spectra showing the dependence of the emission spectra on excitation power.^125^ (F) Spectra of a single 7 μm silica-core CdSe/CdZnS-shell microsphere below (205 μW) and above (366 μW) laser threshold. Inset: Optical and fluorescence micrographs of the microsphere; scale bar indicates 15 μm.^126^ (G) SEM image of a single superparticle of CdSe/CdS nanocrystals.^127^ (H) Emission from a single superparticle of CdSe/CdS nanocrystals at different pump fluences (18–145 μJ/cm^2^) at low spectral resolution (300 lines/mm grating). Inset: High spectral resolution (1800 lines/mm grating), revealing peak substructure.^127^ (I) Time-dependent emission spectra for a superparticles of CdSe/CdS nanocrystals recorded over 15 min of continuous operation at an excitation fluence of 1.6 mJ/cm^2^ before (left) and after (right) light-soaking.^128^ (J) Monodisperse superparticles of CdSe nanocrystals generated using a source-sink emulsion system.^7^ (K) SEM image of a dimer of CdSe/CdS nanocrystal superparticles; (insets) dark-field optical micrographs of superparticles clusters.^7^ (L) Photoluminescence spectra of the clusters shown in (K).^7^ Panels A and B are adapted with permission from ref (123). Copyright 2013 The American Chemical Society. Panel C is adapted with permission from ref (124). Copyright 2002 John Wiley and Sons. Panels D and E are adapted with permission from ref (125). Copyright 2018 The American Chemical Society. Panel F is adapted with permission from ref (126). Copyright 2005 John Wiley and Sons. Panels G and H are adapted with permission from ref (127). Copyright 2018 The American Chemical Society. Panel I is adapted with permission from ref (128). Copyright 2023 The American Chemical Society. Panels J, K, and L are adapted with permission from ref (7). Copyright 2022 The American Chemical Society.

Whispering-gallery modes originate from the presence of rotational symmetry in dielectric objects larger than the wavelength of light. Microfabrication techniques are able to fabricate dielectric structures that are particularly efficient at trapping light through whispering-gallery modes, such as toroidal ring resonators. The precise design and optimized optical response of these resonators can couple to the versatility of colloidal nanocrystals in several ways. As an example, Shi et al. coated a toroidal ring resonator of silica with a polymer film embedded with Au nanorods; see Figure 8A.^123^ By optimizing the diameter of the resonator and the dimensions of the nanorods, the spectral position of a whispering-gallery mode of the resonator is tuned in resonance with the longitudinal plasmonic mode of the nanorods. The evanescent field of the resonant whispering gallery mode is then able to excite the Au nanorods efficiently resulting in lasing; see Figure 8B. The excitation mechanism is revealed to be two-photon excitation,^129^ as shown by the quadratic relationship between input power and photoluminescence intensity, see inset of Figure 8B.

While the use of metallic nanocrystals as emitters represent an upcoming topic of research, using semiconductor nanocrystals as gain medium for lasers is well-established. Currently, the main goal of this field is to reach a practical design for electrically pumped lasers by cutting optoelectronic losses through improved quality of gain material and cavity.^130^ In this respect, whispering-gallery microresonators are peculiar since cavity and gain medium can coincide, resulting in relatively simple designs. One of the earliest reports on whispering-gallery microresonators based on semiconductor nanocrystals uses a cylindrical cavity filled with a dispersion of CdSe/ZnS nanorods representing the gain medium; see Figure 8C.^124^ The excitation light couples through free space to the whispering-gallery modes of the cylindrical cavity, inducing lasing action in the nanocrystal dispersion despite the relatively low photoluminescence quantum yield of of the nanocrystals (14%).

Improvements in cavity design and nanocrystal quality can lead to surprising results. Le Feber et al. designed a whispering-gallery microresonator by engraving a ring-shape in a silicon substrate, filling it with CdSe/CdS/ZnS core/shell/shell nanocrystals, and removing the excess with tape; see Figure 8D.^125^ Increasing the excitation fluence resulted in lasing, where each lasing peak can be associated with a specific mode of the resonator spectrally overlapping with the gain band of the nanocrystals; see Figure 8E. Surprisingly, the authors observed that increasing the excitation fluence resulted in the spectral tunability of the lasing emission from red, to orange, to green. The authors suggest that this phenomenon results from a competition between stimulated emission from the shell and charge-carrier transfer from the shell to the core. When exciting above the bandgap, the shell is responsible for the absorption of light since its volume represents the majority (98%) of that of the nanocrystal. Therefore, the high-energy excitons reside in the shell, from which they transfer to the lower-energy states of the core on a picosecond time scale, resulting in population inversion and red lasing from 1S state of the core. At higher excitation fluences, the occurrence of population inversion in the shell may outpace charge transfer to the core, resulting in the occurrence of green lasing from the shell. Similar results have been reported for spherical superparticles of CdSe/CdS nanocrystals, although there the competition is between the two lowest-energy states in the core alone (1S and 1P).^128^

The spherical shape is probably the most versatile for whispering-gallery microresonators. A high-symmetry geometry, the sphere lends access to a vast number of optical modes, minimizing the need for matching cavity length and spectral position of the optical gain band. Furthermore, since colloidal synthesis is strongly governed by surface tension, the spherical shape enables the use of micron-sized colloids as microresonators. This idea was first demonstrated by Snee et al. when they deposited a layer of CdSe/CdZnS nanocrystals on a low-density film of silica microspheres, see inset in Figure 8F.^126^ After photoexcitation with 400 nm light, the nanocrystal emission couples to the whispering-gallery modes of the microresonators, resulting in population inversion and lasing; see Figure 8F.

Although the idea of using colloidal microspheres as photonic templates is attractive, the mismatch in refractive index between the nanocrystal film and the microcavity can result in unwanted scattering and decrease the efficiency of optical coupling between the photoluminescence and the whispering-gallery modes. This issue can be solved by preparing colloidal microspheres consisting of assembled nanocrystals. By using a droplet microfluidic approach, Montanarella et al. generated colloidal microspheres of highly emissive CdSe/CdS nanocrystals, resulting in colloidal microresonators where the cavity and gain medium coincide, as shown in Figure 8G.^127^ Low-fluence photoexcitation of the superparticles results in an emission spectrum that is typical for these nanocrystals, but higher fluences cause population inversion and multimode lasing; see Figure 8H. High spectral resolution measurements reveal that each lasing peak consists of at least two modes, consistent with the existence of several almost energetically degenerate modes as expected for spherical geometries.

When investigating a comparable system, Neuhaus et al. show that the lasing peaks of CdSe/CdS nanocrystal superparticles are unstable with time, featuring a blue-shift of ∼30 meV over 15 min; see the left panel in Figure 8I.^128^ This instability is highly undesirable for a laser source, where the spectral stability is one of the most coveted and valuable characteristics. The authors attribute the blue-shift to a decrease in the effective refractive index of the superparticles of Δ*n*_eff_ = −0.027 originating from the capture of carriers from trap states, resulting in charging. Interestingly, the authors were able to remove this spectral instability through a light-soaking process consisting of the photoexcitation of superparticles for 10 min with a fluence six times larger than the lasing threshold. After light-soaking, the superparticle lasing appears permanently stable, with a measured blue-shift of less than 0.5 meV; see the right panel in Figure 8I. Likely, the light-soaking protocol results in the complete filling of trap states, which then remain filled at standard conditions for several weeks. In the future, light-soaking may play a role in triggering ligand cross-linking, improving the structural^131,132^ as well as optical stability of superparticles.

The physical properties of the assembled microlasers often bear a strong dependence on their size and shape, creating a need for a reliable method to generate a single size and shape of superparticles. A recent method has achieved this goal by a source-sink emulsion approach; see Figure 8J.^7^ The approach relies on droplet microfluidics to generate monodisperse oil-in-water droplets filled with a nanocrystal dispersion, as the source emulsion. The source emulsion is then mixed with a second nanoemulsion, as the sink emulsion, consisting of oil-in-water droplets. When the size of the sink emulsion is significantly smaller than that of the source emulsion (e.g., 60 nm and 60 μm, respectively), and the water solubility of the oil in the sink emulsion is significantly lower than the oil in the source emulsion (e.g., hexadecane and toluene, respectively), then unidirectional mass transfer of oil from the source to the sink emulsion is observed. Increasing the oil solubility through temperature results in an increase in the rate of mass transfer. This leads to the shrinking of the source emulsion droplets and the efficient formation of nanocrystal superparticles in less than 5 min. The superparticles retain the monodispersity of the initial microfluidic droplets, leading to ∼2% polydispersity.

Drop-casting a suspension of superparticles causes them to assemble into clusters consisting of 1–6 superparticles, as shown in Figure 8K.^7^ When photoexcited, all superparticles clusters show an efficient photoluminescence emission inherited by the constituent nanocrystals. However, the cluster assembled morphology has a significant influence on the lasing behavior of the superparticles. The position of the lasing peaks varies significantly with cluster morphology, suggesting a mechanism for the selection of the modes that participate in lasing; see Figure 8L. Indeed, placing microresonators in series is known to lead to a selection of only those modes with a frequency allowed for all microresonators, resulting in a frequency filter.^133^ Similarly, the small changes in cavity length within the narrow distribution of superparticle sizes constituting the cluster are sufficient to enable optical filtering.

## 3 Conclusions

We have provided a brief review of the emergent properties of 3D assemblies of (nano)particles in confined spaces. Although still in its infancy, this research field provides a fascinating point of contact for physicists, chemists, and engineers. Perhaps, the most significant strength of superparticle science stands in the possibility of achieving a high level of design control over multiple length scales. For example, the presence of chirality over multiple length scales leads to chiral behavior over multiple optical regimes.^101^ Assembling superparticles into even higher order structures may further allow for extended control over the physical properties of the system.^7^ For instance, the structural coloration deriving from functional superparticle assemblies may lead their implementation in photonic conductive or photovoltaic paints.

This level of control over multiple length scales provides a functional bridge between building block synthesis and device implementation. As the synthesis of monodisperse (nano)particles is reaching maturity, the attention of researchers can turn to emergent opportunities in the exploitation of this technology. The interaction between constituent building blocks and the superparticle as a whole is already showing promise, as shown in this review. The acute sensitivity of the photonic modes of the superparticle to local compositional,^134^ environmental,^135^ and mechanical^136^ changes may lead to the development of precise optical sensors. Beyond, the integration of building blocks with orthogonal physical properties (plasmonic, chiral, semiconducting, photonic, insulating, magnetic) promises the development of novel materials with properties that are sensitive not only to the interaction between building blocks and superparticle but also to the local environment of individual building blocks.

Interactions between building blocks can induce order or disorder at the microscale while potentially still displaying the same functional property at the macroscale, for example structural photonic glasses.^118^ This disorder can be valuable as a structural “fingerprint” which is challenging to reproduce. Superparticles with such internal disorder may lead to the development of efficient microscale optical tags for anticounterfeiting.^122^ This technology may be particularly useful and timely in a world where counterfeiting technology, such as deep-fake and artificial-intelligence-powered video technology, is becoming increasingly easy to access and difficult to detect.

By contrast, superparticles with a characteristic internal structure may become the standard to build in specific response functions. For instance, anisotropic superparticles may result in strong electromagnetic fields in a localized region of space; porous superparticles obtained from the removal of one nanoparticle phase may be able to uptake, process, and release cargo at a desired location in response to different external triggers; core/shell superparticles derived from the phase separation of different nanocrystal morphologies may allow for the incorporation of two functionalities on the same superparticle while minimizing cross-talk between the different nanocrystal phases.

Numerous opportunities lie on the path ahead, as well as several challenges. Perhaps the most crucial challenge to solve consists in optimizing the formation of superparticles to achieve a monodisperse product with an efficient methodology. The current state of the art relies on droplet microfluidic approaches in conjunction with the usage of fluorinated chemicals to improve droplet stability during densification^32^ or by the addition of a sink emulsion to increase the rate of densification.^7^ While these approaches work well in producing monodisperse samples, they are characterized by limited sustainability or throughput. Optimizing a method to produce monodisperse samples on gram rather than milligram scale such as continuous emulsification may enable new technologies and implementation in an industrial setting. Furthermore, achieving precise control over the crystallinity of the superparticles without compromising on the final monodispersity of the product would enable a enhanced tunability over their emergent properties. Being able to predict the complex phase behavior of superparticles and the resulting properties *a priori* would be a paradigm shift in their exploitation in farther afield applications.
